# Cellular senescence: at the nexus between ageing and diabetes

**DOI:** 10.1007/s00125-019-4934-x

**Published:** 2019-08-27

**Authors:** Allyson K. Palmer, Birgit Gustafson, James L. Kirkland, Ulf Smith

**Affiliations:** 1 Robert and Arlene Kogod Center on Aging, Mayo Clinic, 200 1st St SW, Rochester, MN USA; 2 Lundberg Laboratory for Diabetes Research, Department of Molecular and Clinical Medicine, Sahlgrenska University Hospital and University of Gothenburg, 413 45 Gothenburg, Sweden

**Keywords:** Ageing, Cellular senescence, Dasatinib, Diabetes, Geroscience, Life course development, Quercetin, Review, Senolytics, Type 2 diabetes

## Abstract

Ageing and diabetes lead to similar organ dysfunction that is driven by parallel molecular mechanisms, one of which is cellular senescence. The abundance of senescent cells in various tissues increases with age, obesity and diabetes. Senescent cells have been directly implicated in the generation of insulin resistance. Recently, drugs that preferentially target senescent cells, known as senolytics, have been described and recently entered clinical trials. In this review, we explore the biological links between ageing and diabetes, specifically focusing on cellular senescence. We summarise the current data on cellular senescence in key target tissues associated with the development and clinical phenotypes of type 2 diabetes and discuss the therapeutic potential of targeting cellular senescence in diabetes.

**Electronic supplementary material:**

The online version of this article (10.1007/s00125-019-4934-x) contains a slideset of the figures for download, which is available to authorised users.

## Introduction: ageing and diabetes are intimately linked

**Fig. 1:**
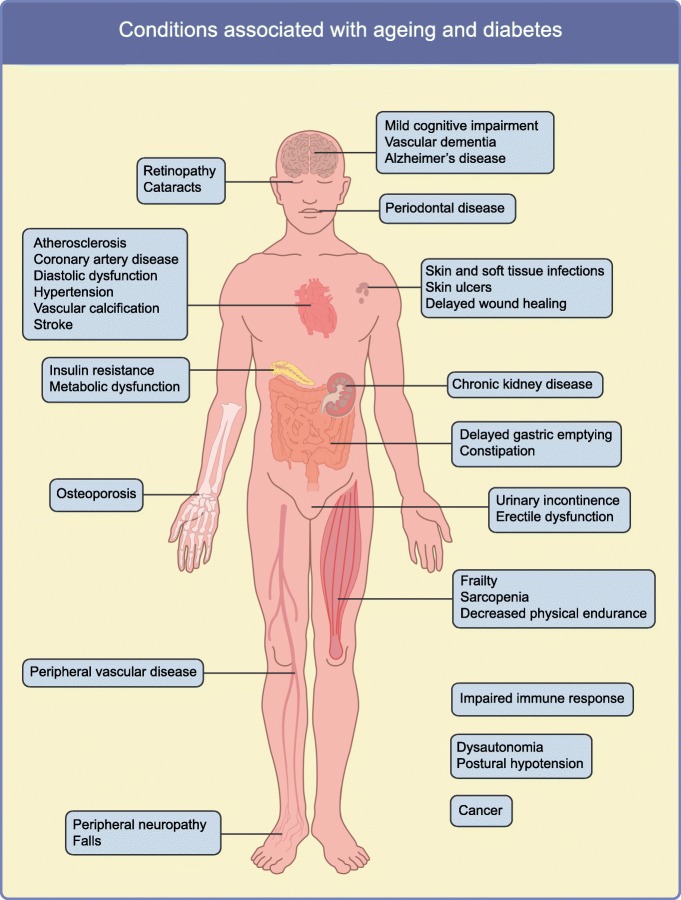
Conditions associated with both advanced age and diabetes. This figure is available as part of a downloadable slideset

The prevalence of diabetes, especially type 2 diabetes, increases with age, with the majority of people in developed countries who have diabetes being over 64 years old [1]. Dysfunction of multiple organ systems manifests similarly in diabetes as it does during normal chronological ageing, but in diabetes this frequently occurs at a younger age [2–4]. Diabetic individuals are more likely to develop age-related comorbidities, such as frailty, mild cognitive impairment, Alzheimer’s disease, cardiovascular disease, bladder dysfunction, osteoporosis, visual impairment and renal dysfunction (outlined in Fig. 1), indicating that type 2 diabetes itself might represent a pro-ageing state.

The geroscience hypothesis leverages the fact that ageing is the major risk factor for most chronic diseases, including diabetes, and predicts that targeting fundamental ageing mechanisms, such as cellular senescence, could have a large impact on increasing health span and decreasing chronic disease burden. This is due to proposed effects on multiple age-related diseases as a group, rather than targeting them individually [5–7].

In this review we explore the biological links between ageing and diabetes, namely, the fundamental mechanisms that may predispose individuals to both age-related and diabetic phenotypes. Specifically, we focus on cellular senescence, a cell fate that occurs in response to cellular stress and involves growth arrest, resistance to apoptosis, and secretion of a host of proinflammatory factors. Evidence is emerging to support that this fundamental ageing mechanism contributes to the development of diabetes, its complications and its comorbidities. In addition, senolytic drugs, which selectively cause apoptosis in senescent cells, have been discovered and have already advanced into clinical trials for other indications. Therefore, we also speculate about cellular senescence as a therapeutic target for future interventions to delay, prevent or treat diabetes and its complications.

## Fundamental ageing mechanisms contribute to diabetes pathogenesis

**Fig. 2:**
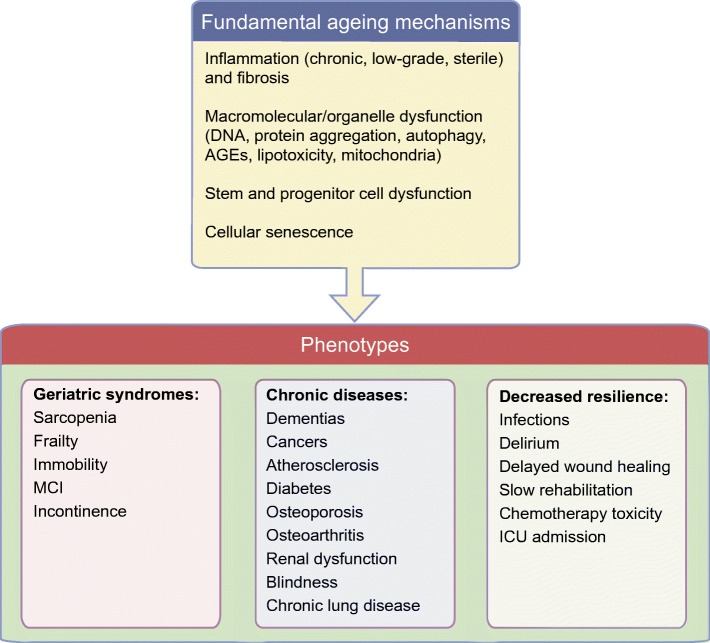
Consequences of fundamental ageing processes. ICU, intensive care unit; MCI, mild cognitive impairment. This figure is available as part of a downloadable slideset

Fundamental ageing mechanisms are many and interrelated. Several frameworks have been described to classify age-associated processes [6, 8, 9], many of which overlap with aberrations seen in obesity and diabetes. This has been particularly well described in the case of adipose tissue [10, 11]. In general, fundamental ageing mechanisms broadly fall into the following categories: (1) macromolecular dysfunction (including loss of proteostasis, failure of DNA damage repair, aberrant mRNA processing); (2) sterile inflammation (infiltration of immune cells and release of proinflammatory cytokines in the absence of a specific pathogen) together with fibrosis; (3) progenitor cell dysfunction (including depletion of progenitor pools, decreased differentiation capacity or aberrant lineage distribution); and (4) cellular senescence (Fig. 2). These categories are discussed in more detail below in the context of their proposed roles in the pathogenesis and progression of diabetes. We place some focus on adipose tissue since it plays a key role in the development of inflammation, insulin resistance and associated disorders, and several mechanistic studies of senescent cells in adipose tissue have been performed.

### Macromolecular dysfunction/damage

Ageing is associated with genomic instability in the form of aberrant gene expression, DNA damage, skewed miRNA processing and epigenetic alterations. In addition to diminishing with age, telomere length is decreased in subcutaneous adipose tissue and leucocytes of obese individuals [12]. Loss of proteostasis has also been described in ageing. For example, markers of endoplasmic reticulum stress are increased and autophagy is decreased in adipose tissue of old vs young mice [13]. Oxidative stress, which increases genomic instability, is increased in obese humans [14]. In addition, AGEs, one consequence of oxidative stress, are a marker of ageing and one of these endproducts is the most widely used measure of glucose control, namely, HbA_1c_ [15].

### Sterile inflammation

Ageing is known to be associated with increased inflammation that is not in response to a particular pathogen. The trigger for this phenomenon, known as ‘inflammageing’, is not known and is likely to be multifactorial, including input from the gut, muscle and liver [16]. However, adipose tissue has been shown to be a major contributor via shifted immune cell populations and inflammatory cytokine release. Similarly, adipose tissue inflammation is strongly implicated in the genesis of insulin resistance in obesity.

There is some evidence that activation of inflammatory pathways also contributes to the insulin resistance seen in old age. For example, aged Toll-like receptor-4 (TLR-4)-deficient mice, which have a diminished inflammatory response, show reduced expression of inflammatory markers and p16^Ink4a^ (also known as cyclin-dependent kinase inhibitor; CDKN2A) in adipose tissue and improved glucose tolerance compared with aged mice with an intact inflammatory response [13]. Changes in cellular composition of adipose tissue with ageing, including a shift of macrophages to a proinflammatory phenotype and loss of T-regulatory cells, are thought to play a role in the genesis of inflammation and associated insulin resistance. Similar changes in cell composition occur in obesity [17].

### Progenitor cell dysfunction

Both ageing and obesity are associated with impaired capacities of adipocyte progenitors to replicate and differentiate into fat cells. Furthermore, impaired adipocyte progenitor function in adipose tissue can independently contribute to insulin resistance [18, 19]. A reduced ability of adipose tissue to expand when challenged with excess nutrients due to decreased adipogenic potential can lead to insulin resistance [20, 21], since this leads to adipocyte hypertrophy, which is associated with increased inflammation, lipolysis and systemic insulin resistance [18, 22, 23]. The high senescent cell burden in hypertrophic obesity may be more a cause of the development of fat cell hypertrophy, rather than being secondary to obesity itself [24]. Reduced adipogenesis as a consequence of increased senescent cell burden is also associated with increased ectopic lipid accumulation in other tissues, such as liver and muscle [24, 25], which in turn is associated with the development of insulin resistance.

### Cellular senescence

Cellular senescence is a cell fate that entails essentially irreversible growth arrest and resistance to apoptosis, which can occur in response to cell stress [26–28]. Several studies in genetically modified animal models have shown that increased cellular senescence leads to pronounced consequences, including dysfunction of multiple organs, reduced physical activity and early mortality [26]. Similarly, transplanting senescent cells, including senescent adipocyte precursors, into young animals induces an ageing-like phenotype with accelerated onset of multiple age-related diseases and early death, while intermittent treatment with senolytic agents, such as the combination of dasatinib and quercetin (D + Q), reverses this [29]. The role of adipose tissue senescence in the pathogenesis of insulin resistance and type 2 diabetes has been well described, as summarised below. Cellular senescence in other tissues, such as the pancreas, muscle and liver is also likely to play an important role in the pathogenesis of type 2 diabetes.

Adipose tissue senescent cell abundance is increased not only with ageing but also in obesity, primarily hypertrophic obesity [19, 30, 31]. Consistent with this, adipose cell size in subcutaneous adipose tissue in non-diabetic individuals is positively related to markers of cellular senescence [31]. In fact, increased senescent cell burden can occur even before type 2 diabetes develops in individuals with a genetic predisposition for the disease. Indeed, polymorphisms in genetic markers of cellular senescence, such as *CDKN2A*, are associated with increased risk for developing both type 2 diabetes and cardiovascular disease [32].

The tumour suppressor p53, a key regulator of adipogenesis, is associated with cellular senescence and needs to be inhibited before adipogenic precursor cells can undergo differentiation into insulin-responsive fat cells [33]. Activation of p53 and accumulation of reactive oxygen species are seen in adipose tissue early during obesity development and, thus, tend to prevent normal adipogenic differentiation. This can even occur before the development of insulin resistance, adipose tissue inflammation and glucose intolerance [34]. Activation of p53 also blunts insulin-induced glucose transport and increases lipolysis in adipocytes, further contributing to both inflammation and insulin resistance [34]. As with ageing, p53 is increased in adipose tissue in type 2 diabetes, and overexpression of p53 in the adipose tissue in animal models leads to systemic insulin resistance [30].

Senescence of adipose progenitor cells is a major negative regulator of adipogenesis, both through cell-autonomous mechanisms and by affecting neighbouring cells via the senescence-associated secretory phenotype (SASP). Once formed, senescent cells can affect the function of neighbouring adipose tissue progenitor cells, inhibiting adipogenesis, as shown in co-culture experiments [35]. In addition, senescent cells can directly cause insulin resistance through secretion of SASP factors such as activin A, IL-6 and TNF-α [35, 36]. Senescent cells also contribute to chemoattraction of macrophages to visceral adipose tissue in obesity [24].

## Senescent cell burden is increased in diabetes

The major risk factors for the development of type 2 diabetes are age and obesity, which are both associated with increased burden of senescent cells. While cellular senescence is postulated to contribute to the development of diabetes, as discussed above, the diabetic microenvironment also seems to lead to increased senescent cell burden. For example, elevated glucose and lipid levels themselves, like inflammation, can induce cellular senescence [11, 37]. Both type 1 and type 2 diabetes are associated with increased risk of glucose-associated microvascular complications involving the eyes, nerves and kidneys. The role of cellular senescence in the pathogenesis of these complications is not well defined.

Although many of the current studies of consequences of cellular senescence have been conducted in animal models, several studies have analysed senescence in human cells from individuals with and without diabetes. Below is a summary of current data on cellular senescence in key target tissues associated with the development and clinical phenotypes of type 2 diabetes.

### Beta cells

The development of insulin resistance necessitates a compensatory increase in insulin secretion for the maintenance of glucose levels, and type 2 diabetes develops when insulin secretion becomes insufficient to overcome the degree of insulin resistance. Several studies have shown that the gene expression pattern in beta cells changes with age and that genes related to cellular senescence, such as *Cdkn2a* and *Cdkn2b*, are increased. Although this leads to a reduced ability to proliferate, a surprising finding was that, rather than being reduced, insulin secretion was actually increased in p16^Ink4a^-induced senescent cells [38]. To what extent this finding can contribute to the increase in basal insulin secretion with age, which is mainly considered to be due to the associated increase in obesity and insulin resistance, is unclear but a provocative concept. Very recently, it was shown that deletion of senescent beta cells in a mouse model of type 1 diabetes enhanced insulin secretion and preserved insulin secretory capacity, providing a novel link between cellular senescence and severe insulin deficiency [39].

### Abdominal/visceral obesity

Inflammation is increased in adipose tissue related to adipocyte hypertrophy, and inflammation can in turn cause accumulation of senescent cells. Obesity itself, probably primarily when associated with hypertrophic expansion, is associated with increased markers of cellular senescence, including adipose tissue β-galactosidase activity, a marker of high lysosomal activity and lysosomal content, as well as increased plasminogen activator inhibitor 1 (PAI-1), p53 and cyclin D kinase inhibitors, including p16^Ink4a^ [30]. Senescent progenitor cells inhibit adipogenesis and promote ectopic lipid accumulation as well as increased visceral fat and abdominal obesity. Similarly, ageing is associated with accumulation of non-dividing senescent cells in adipose tissue [19, 35, 40]. Age-associated increases in visceral fat independent of BMI have also been described [41].

### Fatty liver disease

Type 2 diabetes is associated with increased risk of non-alcoholic fatty liver disease (NAFLD). It has recently been shown that senescent cell burden in the liver is increased in individuals with NAFLD, and that the extent of steatosis correlates with markers of senescence [25]. In mice, induction of senescence specifically in hepatocytes caused increased fat deposition, suggesting a direct role of senescent hepatocytes in the pathogenesis of NAFLD. In addition, steatosis was alleviated by senolytic (D+Q) treatment [25].

### Cardiovascular disease

Cells in the media of the aorta and in atherosclerotic plaques of hypercholesterolaemic (*ApoE*^−/−^) as well as ageing mice show increased markers of senescence. Studies have reported that removal of senescent cells led to improved vascular smooth muscle sensitivity to nitric oxide donors and decreased plaque calcium [40, 42], indicating that senescent cells play a role in endothelial dysfunction in atherosclerosis. In addition, senescent cell clearance in obese mice led to improved cardiac diastolic function, which has translational implications for diabetic patients, in whom heart failure with preserved ejection fraction is common [24]. During the ageing process, senescent cardiac progenitor cells develop a hypertrophic, pro-fibrotic phenotype and undergo loss of replicative capacity. Senescent cell removal in mice alleviated age-related dysfunction of cardiac progenitor cells and decreased fibrotic area formation after myocardial infarction [43, 44].

### Renal dysfunction

Cellular senescence is increased in kidney cells from individuals with type 2 diabetes and increases with age in non-diabetic individuals [45]. The clinical importance of this finding is supported by recent data showing that senolytics (D+Q) alleviated proteinuria in obese insulin-resistant mice [24].

### Cognitive dysfunction and Alzheimer’s disease

Cellular senescence has been shown to play a role in cognitive dysfunction in both obese insulin-resistant mice and in old mice. Senolytic (D+Q) therapy reduced brain senescent cell abundance, restored neurogenesis and alleviated neuropsychiatric dysfunction in obese animals [46]. The same agents reduced neuroinflammation, restored neurogenesis, and partly reversed brain atrophy in old mice with an Alzheimer’s-like state caused by overexpression of Tau protein [47, 48].

Taken together, these findings in both human and animal models strongly support the importance of cell senescence in both the development of diabetes and associated diabetic complications. In addition, they suggest that reducing senescent cell burden may be an important novel therapeutic strategy for diabetes and its complications.

## Ageing and cellular senescence as therapeutic targets in diabetes

Several interventions that target fundamental ageing mechanisms, including, but not limited to, cellular senescence, increase lifespan or improve function in old age in animal models [5, 26, 29, 49–52]. Many of these interventions also delay or prevent progression of disease. The ageing research community is in the process of initiating efforts to translate these findings into human application.

The recent identification of senolytic drugs presents an opportunity for a direct test of the mechanisms by which senescence is involved in diabetes pathogenesis. In diet-induced and genetically-induced obese mice, treatment strategies that reduce senescent cell burden, either via genetic targeting or by administration of senolytic drugs, improve diabetic phenotypes, including glucose tolerance and insulin sensitivity [24]. In addition, these strategies alleviate microalbuminuria and diastolic heart dysfunction [24]. Therefore, senolytics appear to affect multiple tissues when administered systemically. Initial findings suggest that the senolytic approach may be translatable to humans: the first clinical trial of senolytics was published recently and reported that physical function was improved in patients with idiopathic pulmonary fibrosis after 3 weeks of intermittent D+Q treatment [53]. Further study with larger, randomised controlled trials is needed to further evaluate senolytics for the treatment of human disease.

Because senolytics remove senescent cells, rather than preventing their formation, the associated risk of cancer is low. Many senolytic drugs are also used to disable anti-apoptotic pathways in cancers. Indeed, senolytics delay death from cancers in old mice [29]. In addition, the agents are administered intermittently, which may reduce the incidence of side effects. Administration of a single dose of senolytics has been shown to have lasting effects in particular models, such as radiation-induced dysfunction [40]; however, it is likely that the proper dosing schedule will need to be determined for each particular disease state. For example, in diet-induced obesity, one would expect senescent cells to be continually forming because of ongoing metabolic insult, and, therefore, more frequent dosing may be necessary to reduce senescent cell burden.

There are currently gaps in our knowledge regarding the development of clinical strategies to target fundamental ageing mechanisms. Further work is needed to: (1) determine the length of trials needed to fully test such strategies; (2) identify reliable or universal biomarkers of senescence that can be used to assess efficacy of interventions; (3) elucidate the kinetics of senescent cell formation and reappearance, especially in the setting of ongoing insults such as nutrient excess; and (4) assess the efficacy of senolytic treatment in early vs late disease, which may vary by disease state.

Diabetes presents a reasonable situation in which to test senolytic therapies, as multiple pro-ageing mechanisms are at play in diabetes. In particular, complications of diabetes are an excellent outcome to study in clinical trials targeting ageing mechanisms, since few effective disease-modifying interventions are available for these complications, and improvements in function can be readily detected.

## Conclusion

Ageing and diabetes give rise to a similar array of organ dysfunction. Targeting fundamental ageing mechanisms could revolutionise the treatment of diabetes and have a major impact on the prevention of diabetic complications. In particular, senolytic therapies represent a significant opportunity in diabetes, as cellular senescence is intimately linked to diabetes pathogenesis and may also contribute to diabetes progression and the development of diabetic complications.

## Electronic supplementary material

ESM (PPTX 277 kb)

## Abbreviations

D+Q: Dasatinib and quercetin
NAFLD: Non-alcoholic fatty liver disease
SASP: Senescence-associated secretory phenotype
