# Supplementary material for: Cellular senescence: at the nexus between ageing and diabetes
Source: Diabetologia. 2019 Aug 27;62(10):1835–41. doi: 10.1007/s00125-019-4934-x (PMC6731336; doi:10.1007/s00125-019-4934-x)
Supplement: Supplementary file 1 — (PPTX 277 kb) [file 125_2019_4934_MOESM1_ESM.pptx]

## Slide 1
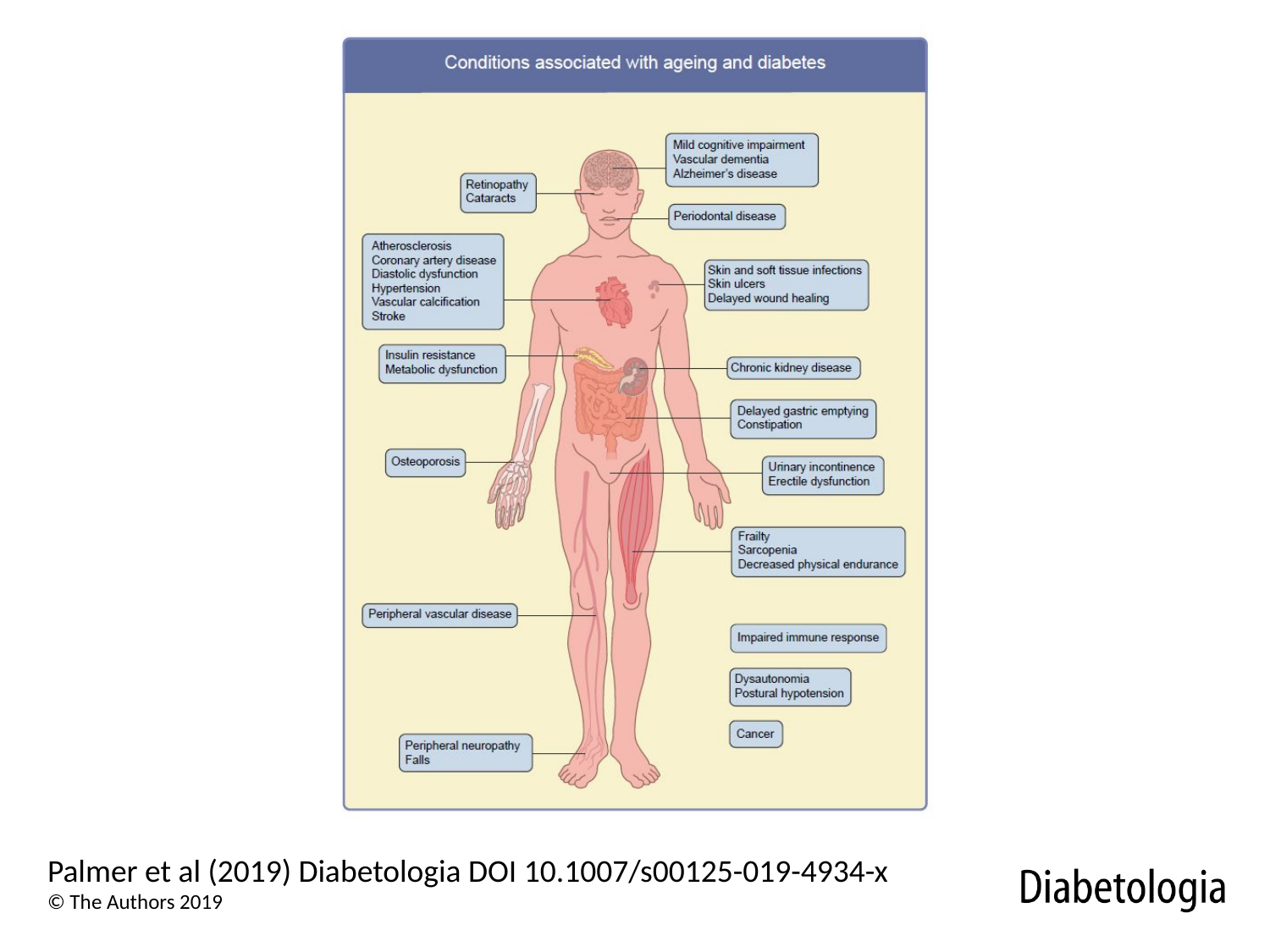

Palmer et al (2019) Diabetologia DOI 10.1007/s00125-019-4934-x
© The Authors 2019

## Slide 2
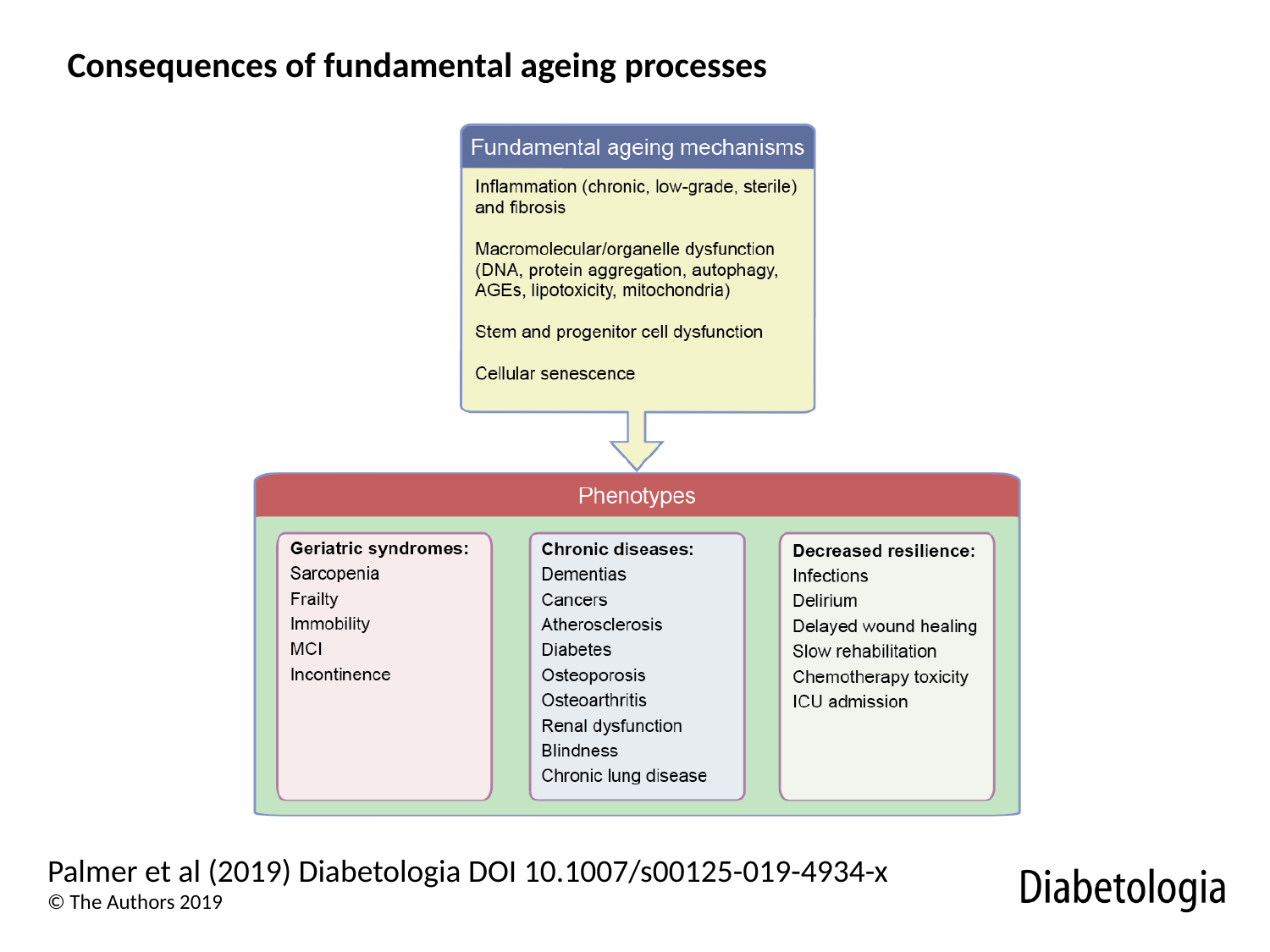

Consequences of fundamental ageing processes
Palmer et al (2019) Diabetologia DOI 10.1007/s00125-019-4934-x
© The Authors 2019
